# Impact of postsurgical complications on hospital costs and margins

**DOI:** 10.1186/cc14272

**Published:** 2015-03-16

**Authors:** R Lavender, M Mythen, J Bao, RH Chapman, F Michard

**Affiliations:** 1 Edwards Lifesciences, Irvine, CA, USA; 2 UCLH National Institute of Health Research, London, UK; 3 Avalere Health, Washington, DC, USA

## Introduction

The impact of postsurgical complications (PSC) on hospital cost has been studied but the impact on margins remains controversial [1]. We assessed economic consequences of PSC in US Medicare patients, and benefits expected from reducing PSC by 14% to 40% with Enhanced Recovery Programs [2].

## Methods

Data from patients with ≥1 comorbidity and major cardiac, vascular, gastrointestinal and orthopedic surgeries in 2011 were extracted from Medicare Standard Analytic Files. Hospital margin was calculated as payment minus cost. Patients with and without PSC were compared, and the economic impact of a 14 to 40% relative reduction in PSC was calculated.

## Results

Of 303,432 patients, 37% had ≥1 PSC. Median length of stay was 10 days for patients with ≥1 PSC and 6 days without (*P *< 0.0001 with vs. without PSC), with readmissions for 21% and 16%, respectively (*P *< 0.0001 with vs. without PSC). Average margins for cases with PSC converted into without PSC would be $1,870 higher. A 14 to 40% reduction in patients with PSC (from 37% to 32% to 22%) would result in saving $153 million to $438 million, and increase hospital margins overall by $28 million to $79 million. See Table 1.

**Table 1:** 

With PSC			Without PSC	
**Mean 2011 US$/patient **	**Cost ($) **	**Margin ($) **	**Cost ($) **	**Margin ($) **
Cardiac	46,535	-2,286	32,887*	-778*
Gastrointestinal	33,280	-3,088	18,942*	-752*
Orthopedic	20,798	-3,567	15,194*	-1,872*
Vascular	31,042	-4,782	17,667*	-2,267*

*P < 0.0001 with vs. without PSC.

## Conclusion

Postsurgical complications have a significant impact on hospital margins. Enhanced Recovery Programs have the potential not only to improve quality of care but also to improve hospital margins.
